# Significant Association of Estrogen Receptor Binding Site Variation with Bipolar Disorder in Females

**DOI:** 10.1371/journal.pone.0032304

**Published:** 2012-02-28

**Authors:** Lisette Graae, Robert Karlsson, Silvia Paddock

**Affiliations:** 1 Department of Neuroscience, Karolinska Institutet, Stockholm, Sweden; 2 Department of Medical Epidemiology and Biostatistics, Karolinska Institutet, Stockholm, Sweden; University of California Los Angeles, United States of America

## Abstract

Major depression is nearly twice as prevalent in women compared to men. In bipolar disorder, depressive episodes have been reported to be more common amongst female patients. Furthermore, periods of depression often correlate with periods of hormonal fluctuations. A link between hormone signaling and these mood disorders has, therefore, been suggested to exist in many studies. Estrogen, one of the primary female sex hormones, mediates its effect mostly by binding to estrogen receptors (ERs). Nuclear ERs function as transcription factors and regulate gene transcription by binding to specific DNA sequences. A nucleotide change in the binding sequence might alter the binding efficiency, which could affect transcription levels of nearby genes. In order to investigate if variation in ER DNA-binding sequences may be involved in mood disorders, we conducted a genome-wide study of ER DNA-binding in patients diagnosed with major depression or bipolar disorder. Association studies were performed within each gender separately and the results were corrected for multiple testing by the Bonferroni method. In the female bipolar disorder material a significant association result was found for rs6023059 (corrected p-value = 0.023; odds ratio (OR) 0.681, 95% confidence interval (CI) 0.570–0.814), a single nucleotide polymorphism (SNP) placed downstream of the gene coding for transglutaminase 2 (TGM2). Thus, females with a specific genotype at this SNP may be more vulnerable to fluctuating estrogen levels, which may then act as a triggering factor for bipolar disorder.

## Introduction

Women are diagnosed with mood disorders much more frequently than men. As a consequence, the reported lifetime prevalence rate in women for developing major depression is nearly twice as high compared to men. In the Netherlands Mental Health Survey and Incidence Study (NEMESIS) and the American National Comorbidity Survey (NCS), two large cross-sectional population studies with comparable designs, for example, the female to male ratio was 1.8 and 1.65, respectively [1]–[4]. Both studies used the Diagnostic and Statistical Manual of Mental disorders, 3^rd^ edition, revised (DSM-III-R) as diagnostic criteria and the Composite International Diagnostic Interview (CIDI) as the psychiatric interview. NEMESIS reported a life-time prevalence for major depression in women of 24.5%, which is roughly equivalent with the 21% reported for women in the NCS study. For males, the life-time prevalence was reported to be 13.6% in the NEMESIS study and 12.7% in the NCS study. Whether the reported gender difference in prevalence is due to biological differences (e.g. differences in endocrine levels) or other factors (e.g. social status discrimination, social stress or gender differences when it comes to reporting symptoms) has for long been a matter of debate [3], [5], [6]. For bipolar disorder (also known as manic depression), the prevalence rate is equal in women and men (1%) [7]–[9]. Biological differences in the etiology may still exist, since it has been reported that depressive episodes may be more common in female compared to male bipolar patients [8], [10]. Moreover, periods of hormonal fluctuations (e.g. premenstrually, postpartum and postmenopausally) have in numerous studies been reported to be associated with increased risk of affective dysregulation and vulnerability to develop depression [11]–[14]. Postpartum depression occurs in up to 10–20% of mothers in the year following delivery [15]. For women with a previous diagnosis of a mood disorder, the risk is even higher with reported rates of approximately 20–30% for women with major depression and as high as 25–52% for women diagnosed with bipolar disorder [15], [16]. Postmenopausal women, with reduced estrogen levels, benefit less from antidepressant treatments compared to premenopausal women [17].

In addition to regulating reproduction and modulating sexual behavior, estrogen, one of the primary female sex hormones, affects several other systems in the body such as the cardiovascular, musculoskeletal, immune and central nervous systems [18], [19]. Estrogen mediates its effects mostly by binding to estrogen receptors (ERs). There are two different types of ERs: one is a member of the nuclear hormone family of intracellular receptors and one is a membrane bound G-protein coupled receptor. This study focuses on the role of the nuclear hormone receptor, a DNA binding transcription factor involved in gene regulation and expression. Two forms of this type of the receptor occur: ERα and ERβ. In order to bind to DNA, this receptor forms either homodimers (α/α or β/β) or heterodimers (α/β).

The DNA binding of ERs is either direct, where the dimer itself binds to specific DNA sequences (so called Estrogen Response Elements, EREs) or through a tethered pathway where the ER dimer binds to other transcription factors (like AP-1 and SP-1) which bind to DNA sequences lacking EREs. In either case, ER binding has an effect on gene transcription. The derived minimal consensus ERE sequence is a 13 base pair palindromic inverted repeat: 5′-GGTCAnnnTGACC-3′, where n is any nucleotide [20]–[22]. Many functional ERE-sequences vary from the consensus by one or more nucleotides. However, it has also been shown that a single nucleotide change may completely disrupt the ER-binding ability [20].

Since ERα and ERβ function as transcription factors by binding to specific DNA sequences and regulate gene transcription, a nucleotide change in the ER-binding sequence could possibly affect the binding and lead to abnormal function. This might alter transcription levels of nearby genes, which could have an effect on the cell function and could be part of a disease mechanism. The aim of this study was therefore to carry out association studies between ER DNA-binding sequence polymorphisms in women or men with bipolar disorder or major depression compared to healthy controls.

Several studies have investigated a possible involvement of ERs in the etiology of psychiatric disorders. To our knowledge, all of them have had a different approach than employed in this study, investigating the genomic areas of the ERα and ERβ genes themselves. Two studies of possible involvement of the ERα- and ERβ-genes in bipolar disorder could not find any association with disease [23], [24]. However, Weickert et al [25] reported that differences in the ERα gene and its mRNA expression contributed to risk for developing schizophrenia, and another study found that persons committing suicide had decreased levels of ERβ mRNA [26].

To our knowledge, no one has so far conducted a systematic and genome-wide study of differences in ER-DNA binding sequences in mood disorders. We therefore designed a study to search for such differences in patients diagnosed with major depression or bipolar disorder. Due to above-mentioned differences in the prevalence and etiology of mood disorders between women and men, we decided to carry out analyses for each gender separately. Correction for multiple testing was carried out using the Bonferroni method.

## Materials and Methods

### Material

#### Study population

Genome-wide genotype data for patients diagnosed with either bipolar disorder or major depression and healthy controls were obtained from the Genetic Association Information Network (GAIN). Genotype data for 964 cases (487 women and 477 men) diagnosed with bipolar disorder and 998 healthy controls (490 women and 508 men) as well as 1727 cases (1200 women and 527 men) diagnosed with major depression and 1758 controls (1076 women and 682 men) were included in the final data set for the association analyses.

The individuals included in the study on bipolar disorder come from the National Institute of Mental Health Human Genetics Initiative (NIMH GI; http://nimhgenetics.org/). Cases used within our study are individuals with European Ancestry that have been collected and characterized by the NIMH GI for Bipolar Disorder Consortium (Bipolar consortium). The cases were diagnosed with a standard best estimate final diagnosis (BEFD) procedure and interviewed with the Diagnostic Interview for Genetic Studies (DIGS). The control subjects, also of European ancestry, were collected separately through a NIMH-supported contract mechanism between Dr. Pablo Gejman and Knowledge Networks, Inc. Individuals were excluded from the study after the quality control analyses if they failed any of several quality metrics, such as: low call rate, excessively high or low heterozygosity, incompatibility between reported gender and genetically determined gender, or unexpected familial relationships [9].

For the major depression study, the cases came from the Netherlands Study of Depression and Anxiety (NESDA; http://www.nesda.nl), which is a longitudinal cohort study. Inclusion criteria were a lifetime diagnosis of DSM-IV MDD as diagnosed by the Composite International Diagnostic Interview psychiatric interview, age 18–65 years, and self-reported western European ancestry. The cases were recruited from mental health care organizations, primary care, and community samples. The control subjects were derived from the Netherlands Twin Register (NTR; http://www.tweelingenregister.org), which has collected longitudinal data from twins and their families since 1991. Individuals that did not meet quality control criteria were removed from the study. Such criteria could be: individuals with uncertain linkage between genotype and phenotype, individuals with genomic outliers, such as too high genome-wide homozygosity (∼75%), samples with contamination or individuals that either failed genotyping or had had excessive missing genotype data (<25%) [27].

#### Ethics Statement

Written informed consent had been obtained by the original investigators from all participants in the study. The study was conducted in accordance with data use certification agreements between the Genetic Association Information Network (GAIN) and the investigators.

#### Single Nucleotide Polymorphisms (SNPs) included in our study

The bipolar disorder samples were genotyped by the Broad Institute Center for Genotyping and Analysis (http://www.broad.mit.edu/node/306) and has been described by Smith et al [9]. The Affymetrix Genome-Wide Human SNP Array 6.0 platform was used for the genotyping. Briefly, the initial quality control removed SNPs if the minor allele frequency was <0.01, the call rate was <95%, if the SNP had a Hardy-Weinberg Equilibrium p-value of <1×10^−6^ in the control sample, if there were three or more Mendelian errors in HapMap trios included in the run for quality control purposes, or if there was more than one discrepancy among duplicate samples. Of the 729 454 SNPs that passed the initial QC analyses in the bipolar disorder study, 724 067 SNPs were included in the dataset obtained through dbGAP (database of Genotypes and Phenotypes). Study Accession id for the bipolar study is phs000017.v3.p1.

The genotyping of the major depression sample was conducted by Perlegen Sciences (Mountain View, CA, USA) and has been described elsewhere [28]. The Perlegen GWAS platform used for the analysis contained 599 156 SNPs. Briefly, the initial quality control analyses removed SNPs with the following features: gross mapping problem, ≥2 genotype disagreements in 40 duplicated samples, ≥2 Mendelian inheritance errors in 38 complete trio samples, minor allele frequency <0.01 or >0.05 missing genotypes in either cases or controls. A Hardy-Weinberg filter was not used. A total of 435 291 SNPs survived the initial quality control analyses and were included in the released data set (dbGaP Accession Study phs000020.v2.p1).

## Methods

Perl scripts were written for all data manipulation and PLINK, a free, open-source whole genome association analysis toolset (http://pngu.mgh.harvard.edu/~purcell/plink/), was used for additional quality control steps, association analysis, and statistical calculations.

SNPs located in ER binding regions were identified by two approaches: a) the use of publicly available genome-wide Chromatine Immuno Precipitation (ChIP) data on ERα and ERβ binding regions, and b) Estrogen Response Element (ERE) sequence information. In order to have sufficient confidence in a candidate polymorphism, we decided to include into the study 1) those polymorphisms that were found in regions consistently implicated by functional studies (whether or not a predicted ERE binding site was present) and 2) those polymorphisms that were found to be located in *in-silico* predicted binding sites that were validated by at least one functional study.

### A) Identifying SNPs in ChIP-regions

Genome wide Chromatine Immuno Precipitation (ChIP) results of ER DNA-binding were obtained from three sources: [29]–[31].

The three studies differed regarding ER-subtype studied, ChIP method used and number of ER-binding sites identified. Carroll et al studied the ERα by regular ChIP technique and found 3 655 binding sites, while Lin et al also studied the ERα but with a ChIP-PET method and identified 1 234 binding sites. Zhao et al studied the ERβ with regular ChIP technique and identified 1 457 binding sites. In our study we included SNPs located within 343 regions where the three ChIP studies agreed with each other.

### B) Identifying SNPs in ERE-sequences

After downloading IUPAC masked FASTA-files (Mar. 2006(hg18) SNP129) from the UCSC Genome Browser website (http://genome.ucsc.edu/), we searched through the genome for ERE-halfsite core sequences (GGTCA or TGACC) that contained SNPs. We then sought additional support for this in-silico prediction from the ChIP studies and included those ERE binding site disrupting SNPs that were in regions supported by any of the three ChIP-studies.

### Identifying SNP genotypes

For interesting SNPs identified by the methods above but not genotyped in the GAIN data, we tried to find other, genotyped SNPs that were in high Linkage Disequilibrium (LD) (r^2^>0.89). HapMap linkage disequilibrium data (HapMap population CEU, HapMap_rel23a_NCBI_Build 36) was downloaded (http://hapmap.ncbi.nlm.nih.gov/), allowing us to identify markers in high LD with SNPs selected for our study.

### Data management and statistics prior to association analyses

In addition to the quality control performed by the original investigators of the two GAIN studies, we performed a few further quality control steps and excluded SNPs with missing genotypes >0.05, minor allele frequencies <0.01, and Hardy-Weinberg Equilibrium (HWE) p-value of <0.001 in the controls. Individuals were excluded from the study if the missing genotype rate/person was >0.1.

### Association studies

A two-tailed Fisher's exact test was used to study associations between the case and control groups. Association analyses were performed separately in each disease and for each gender. Since two independent populations (female or male) and two independent datasets (major depression or bipolar disorder) were used, Bonferroni correction was appropriate, correcting for a total number of four independent hypotheses and 225 SNPs. Each p-value was thus multiplied by 900 (2×2×225) to give us the Bonferroni-corrected p-values.

## Results

### SNPs included in our study

**Figure 1:**
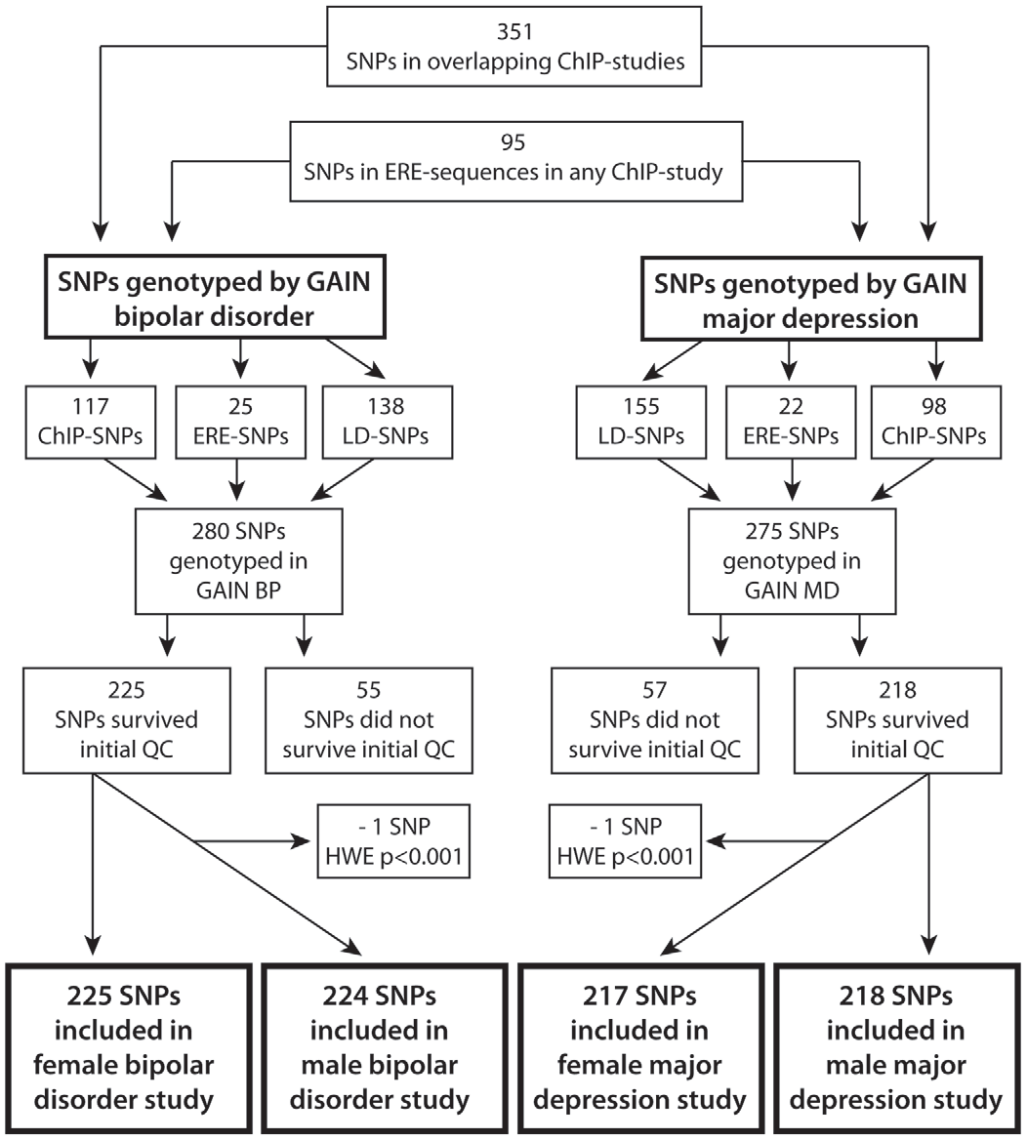
Flowchart illustrating the selection and inclusion process of the SNPs included in our study.

A flowchart of the selection and inclusion process of the SNPs included in our study is shown in Figure 1. A total of 351 SNPs were identified to be located within regions where the three experimental ChIP-studies overlapped. Of these, 117 were genotyped in the GAIN bipolar disorder study and 98 were genotyped in the GAIN major depression study. Additionally, 95 SNPs were found to be placed in ERE-halfsite sequences within any of the three ChIP-studies, and they were thus also included in our study. 25 of these SNPs had been genotyped in the bipolar disorder study and 22 of them had been genotyped in the major depression study.

For the selected SNPs not genotyped by GAIN we tried to find another SNP in high LD (r^2^ = 0.89) with the SNP of interest that was included in the GAIN study.138 such SNPs were added to the bipolar disorder study and 155 high LD SNPs were included in the major depression sample. In total we thus had 280 SNPs for the bipolar disorder study and 275 SNPs for the major depression study that were either identified in all three ChIP-studies or placed in a ERE-sequence within any of the three ChIP-studies. These SNPs had either been genotyped in the GAIN material or were in high LD with a SNP included in the GAIN material.

After quality control, the final datasets for the association analyses included 225 SNPs in the female bipolar disorder sample and 224 in the male sample. The major depression study contained 218 SNPs in the male analysis and 217 SNPs in the female analysis.

### Association analyses

The complete association results can be seen in Tables S1, S2, S3, and S4. Results for the top-ranking SNPs in each study are summarized in Table 1. After correction for multiple testing, rs6023059 showed statistically significant association with disease (corrected p-value = 0.023; odds ratio (OR) 0.681, 95% confidence interval (CI) 0.570–0.814) in the female bipolar disorder material. In the male bipolar disorder sample as well as in the major depression samples the association results for this SNP were non-significant. This SNP is in high LD (r^2^ = 0.89) with rs909859, a SNP located in a region identified by all three ChIP-studies. Both SNPs are placed in the downstream genetic region of two genes, see Table 2 describing the genomic locations of the SNPs identified in the association analyses. The first gene codes for Transglutaminase 2 (TGM2) and is located on the genomic reverse strand. The second gene is called RPRD 1B, located on the forward strand. The TGM2 gene has previously shown to be implicated in neurodegenerative diseases [32] and has been shown to be regulated by estrogen in numerous studies, see Table 3 for the function of the identified SNPs and their estrogen regulations [33]–[35].

**Table 1:** Association study results.

disease	gender	rs-number	alleles^1^	MAF^2^ (cases)	MAF^2^ (controls)	p-value	corrected p-value^3^	OR^4^	L95^5^	U95^6^
bipolar disorder	female	rs6023059	C<T	0.4251	0.5204	0.00002509	0.023	0.681	0.570	0.814
bipolar disorder	female	rs867286	A<G	0.2922	0.3722	0.0001777	0.160	0.696	0.576	0.842
bipolar disorder	male	rs6517590	A<G	0.4906	0.4270	0.005008	>1	1.292	1.082	1.543
major depression	female	rs7217437	C<T	0.4783	0.4345	0.00318	>1	1.193	1.062	1.342
major depression	male	rs16848941	C<T	0.1613	0.1081	0.000155	0.140	1.587	1.251	2.012

1 minor allele<major allele.

2 minor allele frequency.

3 p-value corrected with Bonferroni correction.

4 estimated odds ratio for the minor allele.

5 lower bound of 95% confidence interval for odds ratio.

6 upper bound of 95% confidence interval for odds ratio.

**Table 2:** Genomic information for the top associated SNPs.

disease	gender	rs-number	chr^1^	position (bp)^2^	nearby gene	SNP position relative to nearby gene
bipolar disorder	female	rs6023059	20	36171185	TGM2: transglutaminase 2 and RPRD 1B	56 kb downstream of TGM2 and 17 kb downstream of RPRD1B
bipolar disorder	female	rs867286	2	45835534	PRKCE: protein kinase C, epsilon	intron 1
bipolar disorder	male	rs6517590	21	40618105	DSCAM: Down syndrome cell adhesion molecule	intron 21
major depression	female	rs7217437	17	62894224	PITPNC1: phosphatidylinositol transfer protein, cytoplasmic 1	intron 1
major depression	male	rs16848941	2	164384159	GRB14: Growth factor receptor binding protein 14	673 kb downstream of GRB14

1 chromosome.

2 base pair.

**Table 3:** Function of genes proximal to the SNPs identified in the association studies.

disease	gender	rs-number	nearby gene	function	estradiol regulation	previously associated with mood disorders
bipolar disorder	female	rs6023059	TGM2: transglutaminase 2	Calcium dependent enzyme that catalyzes crosslinking of proteins. Involved in cytoskeleton stabilization, apoptosis, cell differentiation, cell adhesion and GTP-hydrolysing activity.	down regulation/decreased activity	yes
bipolar disorder	female	rs6023059	RPRD1B	Involved in regulation of nuclear pre-mRNA domain containing.	unknown	no
bipolar disorder	female	rs867286	PRKCE: protein kinase C, epsilon	Involved in neuron channel activation and apoptosis. Protein activated by calcium and DAG.	down and up regulation depending on the stimulation context within the cell	yes
bipolar disorder	male	rs6517590	DSCAM: Down syndrome cell adhesion molecule	Involved in Down syndrome, cell adhesion and neuronal development.	unknown	yes
major depression	female	rs7217437	PITPNC1: phosphatidylinositol transfer protein	Cytoplasmic protein that transfers phosphatidylinositol from one membrane compartment to another.	up regulation/increased activity	yes
major depression	male	rs16848941	GRB14	Adaptor protein that interacts with tyrosin-kinases.	down regulation/decreased activity	no

None of the other SNPs reached significance after correcting for multiple testing, but several of the top results (including the second-ranking SNP in the female bipolar disorder material) were obtained in interesting genetic regions previously implicated in the pathogenesis of psychiatric disease. Two of the identified SNPs, rs7217437 and rs867286, are placed in the first intron of genes (PITPNC1 and PRKCE respectively) involved in the inositol pathway. In numerous studies this pathway has been linked to bipolar disorder. Furthermore, lithium, the primary therapeutic treatment for bipolar disorder, works regulatory on this pathway [36]–[38]. The marker rs6517590 is placed in intron 21 in the gene coding for Down syndrome cell adhesion molecule (DSCAM), which is highly important for neural development and contributes to the disease mechanisms in Down syndrome.

## Discussion

To our knowledge, this is the first study to examine the possible involvement of differences in ER-DNA binding sequences in mood disorders. After correcting for multiple testing, a significant association was found for rs6023059 (corrected p-value = 0.023; OR 0.681, 95% CI 0.570–0.814) in the female bipolar disorder material. Thus, our results suggest that this variation in ER-binding sequence may influence risk to become affected by bipolar disorder in women.

Studies reporting a gender difference in the phenotype of depression suggest that these differences might be coupled to differences in estrogen levels and hormonal fluctuations (for review see [14]). Several studies have shown that periods of depression often correlate with hormonal fluctuations in women with bipolar disorder or major depression [10]–[16]. Both these disorders are highly heritable diseases with a complex genetic pattern and a substantial polygenic component. In addition it has previously been suggested that there is also a gender difference in the genetic component of mood disorders [39], a hypothesis our results support.

Genome-wide association studies offer a powerful tool to study genetically complex diseases. The first-pass analysis usually includes all markers after quality control and demands significant results to survive genome-wide correction for multiple testing. However, this correction may be over-conservative. While, given the currently available still modest sample sizes, few genetic association results may thus be strong enough to overcome the multiple testing problem when studying the entire dataset, there may be other, more focused hypotheses that can be tested in the same data. In this way, the multiple-testing problem can be overcome to some extent by a smaller number of SNPs tested within such hypothesis-driven approaches. Genes with a modest influence on disease risk may be identified. Needless to say, there are an infinite number of such possible hypotheses, and replication in independent datasets remains crucial before definite conclusions can be made.

After correction for multiple testing, a significant association result was found for rs6023059 in the female bipolar disorder material. This SNP is placed in the genomic interval between the genes RPRD1B and TGM2. TGM2 is a ubiquitously expressed, highly regulated multifunctional protein. When calcium-bound, this enzyme has a cross-linking activity forming ε(γ-glutamyl)lysine bonds within or between various proteins. TGM2 has been shown to be important for several different cellular processes such as cytoskeleton stabilization, apoptosis, cell differentiation, cell adhesion, and even GTP-hydrolysing activity. TGM2 activity is also well known to be involved in celiac disease (CD), a chronic, auto-immune mediated, small-intestinal disorder characterized by inflammatory injury to the mucosa following ingestion of wheat gluten [40]. TGM2-specific antibodies are almost exclusively found in CD and are therefore used as a diagnostic indicator of CD [41]. In addition, TGM2 has also been implicated in the etiology of several neurological disorders such as Huntington's disease, Alzheimer's disease (AD), Parkinson's disease (PD) and schizophrenia [32], [40], [42]–[45].

Apparently, some women are more vulnerable to normal hormonal fluctuations (eg premenstrually, during the postpartum period and perimenopausally) than others. In these women, a drastic decrease in estrogen levels might increase the risk for experiencing anxiety and depressive symptoms [46]. Reduced levels of estrogen has been shown to increase the activity of the TGM2 protein, something Assisi et al could see when studying apoptosis mediated by TGM2 in the frog liver [33]. In concert with this Fujita et al showed a distinct reduction of the activity of the TGM2 enzyme after estrogen administration, something which inhibited the apoptotic processes in the hippocampus mediated by the TGM2 enzyme [35]. Increased apoptotic activity in patients diagnosed with bipolar disorder compared to healthy controls has been reported in numerous studies [47]–[50]. Moreover, it seems that one of the effects seen by lithium is reduced neuronal apoptosis [51]–[53]. In addition, lithium has an effect on intra cellular calcium homeostasis, which in many cases have been shown to be impaired in bipolar patients [54]–[56]. Calcium is required for TGM2 to be able to adopt a catalytically active conformation. Thus, a perturbed calcium homeostasis, as seen in many bipolar patients, might result in an inappropriate activation of the enzyme [32].

Numerous studies have shown that estrogen can exert an anti-inflammatory effect in the brain. Several in vivo studies on lipopolysaccaride (LPS) induced inflammation in microglia and astrocytes have shown that estrogen administration reduces microglia as well as astrocyte reactivities and down regulates the release of nitric oxide (NO) and several pro-inflammatory cytokines, such as tumor necrosis factor α (TNF-α) and IL-6 [57]–[60]. The NO synthesis in activated microglia has been shown to correlate with an increase of the TGM2 protein expression. Moreover, inhibition of the TGM2 enzyme can reduce the NO secretion in a dose-dependent manner [61]. The TGM2 protein is expressed throughout the body and most notably in the brain. One may thus speculate that immunological reactivity against TGM2 in vulnerable persons may trigger an inflammatory response which may cause damage to neurons [40].

The second top-SNP in the female bipolar material is placed in intron 1 in a gene coding for protein kinase C epsilon (PRKCE). This protein modulates neuronal calcium signaling by binding to specific voltage-gated calcium channels expressed in the axons [36], [62], [63]. Several studies have reported increased levels of basal calcium levels in blood samples from patients with bipolar disorder and it has been suggested that dysregulation of intracellular calcium homeostasis may be involved in controlling mood states in this disease [36]. Lithium, the primary therapeutic choice for bipolar disorder, has been shown to decrease the levels of PRKCE, a mechanism that could be involved in the therapeutic effect of this drug [36], [64].

In the male bipolar disorder study the best association result was received for a SNP placed in intron 21 in the gene coding for Down syndrome cell adhesion molecule (DSCAM) a gene known to be involved in Down syndrom. DSCAM is important for neural development and is involved in axon guidance, neural differentiation and neural plasticity [65], [66]. A possible involvement of the DSCAM gene in bipolar disorder has previously been reported [65].

In the female major depression sample the top-SNP is placed in intron 1 in the gene phosphatidylinositol transfer protein (PITPNC1), a cytoplasmic protein that transfers phosphatidylinositol and phosphatidylcholine between cellular membranes. The mood-stabilizing drug lithium has been shown to down regulate PITPNC1 mRNA expression levels in the mouse brain [67].

The top-SNP in the major depression male material is placed downstream of the gene growth factor receptor binding protein 14 (GRB14), which is a protein that performs both adaptor and modulatory roles in receptor tyrosine kinase signaling.

It has been shown that the estrogen receptor DNA-binding is distributed within and between genes rather than being restricted to promoter-proximal regions. Only a small portion (5%) of ER binding sites are within 5 kb of the transcriptional start site of a gene. The majority (38%) of the binding places are instead located intragenic to the introns of genes whereas functional ER-binding sites are rarely seen in the exons. Furthermore, the ER binding is not limited by binding site orientation and about equal amount of binding occurs within 100 kb from the 5′ start site (23%) as within 100 kb of the 3′ polyadenylation site (19%) [29], [30].

All of the identified SNPs, except rs6517590, are placed either within or close to genes that previously have been shown to be regulated by estrogen (see Table 3). The effect on the gene and/or protein expression varies from inhibiting, as for TGM2 and GRB14, to enhancing, as for PITPNC1, or both, as for PRKCE, where the effect depends on the stimulation context within the cell [33]–[35], [68]–[71].

One limitation of our study is that ER-binding data is derived from one single cell-line type and that relevance of the identified binding sites elsewhere remains to be established. Furthermore, functional studies need to be carried out to confirm influence of polymorphism on ER-activity. Finally, as always in complex genetics, our results need to be replicated in other materials before final judgment.

In conclusion, we performed the first study on ER-DNA binding variation in mood disorders. Due to previously reported differences in the prevalence and etiology of mood disorders between women and men, we studied each gender separately. Our results suggest that there is a gender difference in the genetic pattern contributing to the susceptibility of developing these disorders and that differences in estrogen receptor binding might contribute to a risk of developing bipolar disorder in women.

After correcting for multiple testing, a significant association was found for rs6023059, placed in the downstream genomic region of TGM2, in women with bipolar disorder. Thus, fluctuating levels of estrogen as a triggering factor in combination with specific variants in this estrogen interacting gene may be involved in the disease etiology of bipolar disorder in women.

## Supporting Information

Table S1 **Association results for the female bipolar disorder material.**
(XLS)

Table S2 **Association results for the male bipolar disorder material.**
(XLS)

Table S3 **Association results for the female major depression material.**
(XLS)

Table S4 **Association results for the male major depression material.**
(XLS)
